# The Big Minority View: Do Prescientific Beliefs Underpin Criminal Justice Cruelty, and Is the Public Health Quarantine Model a Remedy?

**DOI:** 10.3390/ijerph22081170

**Published:** 2025-07-24

**Authors:** Alan C. Logan, Susan L. Prescott

**Affiliations:** 1 Nova Institute for Health, Baltimore, MD 21231, USA; susan.prescott@uwa.edu.au; 2 The Medical School, University of Western Australia, Perth, WA 6009, Australia; 3 Family and Community Medicine, University of Maryland, Baltimore, MD 21201, USA

**Keywords:** public health quarantine, social determinants, mass incarceration, punishment, developmental origins, environment, legalome, justice

## Abstract

Famed lawyer Clarence Darrow (1857–1938) argued strongly for an early-life public health approach to crime prevention, one that focused on education, poverty reduction, and equity of resources. Due to his defense of marginalized persons and his positions that were often at odds with his legal colleagues and public opinion, he was known as the Big Minority Man. He argued that the assumption of free will—humans as free moral agents—justifies systems of inequity, retributive punishment, and “unadulterated brutality.” Here, the authors revisit Darrow’s views and expand upon them via contemporary research. We examine increasingly louder argumentation—from scholars across multiple disciplines—contending that prescientific notions of willpower, free will, blameworthiness, and moral responsibility, are contributing to social harms. We draw from biopsychosocial perspectives and recent scientific consensus papers calling for the dismantling of folk psychology ideas of willpower and blameworthiness in obesity. We scrutinize how the status quo of the legal system is justified and argue that outdated notions of ‘moral fiber’ need to be addressed at the root. The authors examine recent arguments for one of Darrow’s ideas—a public health quarantine model of public safety and carceral care that considers the ‘causes of the causes’ and risk assessments through a public health lens. In our view, public health needs to vigorously scrutinize the prescientific “normative” underpinnings of the criminal justice system.

## 1. Introduction

“*We may isolate the criminal of the future as we isolate the typhoid patient of today, not with hatred and fear, but with kindliness and trust in recovery*”

Clarence Darrow, 1928 [1]

Much has been written about Clarence Darrow (1857–1938), oft-celebrated as one of the most impressive lawyers of the 20th century. He is best known as “the attorney for the damned” or the “big minority man”, a lawyer who paired his defense of the underdog with his scorn for gross socioeconomic inequalities and widening power differentials in American society [2,3]. Darrow argued strongly for an early-life public health approach to crime prevention, one that focused on education, poverty reduction, and equity of resources [4,5]. He maintained that in the courts and carceral systems, folk psychology beliefs in moral fiber and willpower are the steel girders that otherwise allow for inequity, retributive punishment, and “unadulterated brutality” [6]. While still a relative minority, scholars from the fields of biology [7], medicine [8], psychology [9], law [10], and philosophy [11], are challenging folk psychology ideas of willpower and dissecting the enduring justifications for retributive punishment.

Here in this viewpoint article, the authors examine the role of folk psychology ideas in justice systems and scrutinize the ways in which the prescientific structures of retributive court systems are defended. Next, we draw from obesity sciences, and recent international consensus papers calling for the dismantling of folk psychology ideas of willpower and blameworthiness. Obesity, we argue, represents an unambiguous example of the ways in which folk psychology and ‘moral fiber’ beliefs contribute to social harms. It also provides a point of entry for the ways in which transdisciplinary research is challenging outdated folk psychology ideas. We then look to the public health quarantine model, a non-retributive approach to justice that accepts constraints on human agency, while considering the ‘causes of the causes’ and risk assessments through a public health lens [12]. Finally, we address the fallacy of Phineas, the common but unscientific idea that brain-related ‘excuses’ for criminal behavior require obvious tissue destruction. We argue that public health is well positioned to scrutinize the widening gulf between scientific knowledge and the rigid prescientific structure of criminal justice.

## 2. Folk Psychology, Willpower, and the Courts

At the outset, the authors approach the term free will in a way that has been described as mainstream or popular: that is, in a given situation and notwithstanding relatively rare exceptions, humans have a universal capacity to choose to behave in any one of many different ways [10]. This ability to control choices and outcomes in the face of multiple possibilities is at the heart of moral responsibility [13]. While this explanation of free will bypasses the esoteric and often confusing discourse that occurs within the field of philosophy [10], it is the foundation upon which the justice system rests.

While the existence of free will has been the subject of rich discourse in philosophy, a personal belief in free will—with its associated convictions and subjective feelings—is a psychological construct. Research shows that belief in free will at the individual level has been associated with a variety of positive outcomes, including academic outcomes [14], job performance [15], wellbeing [16], and meaning in life [17]. Setting aside the difficulty of capturing the nuances of various stances on free will [18], for now, we can accept that free will beliefs are good for the self. The more debatable issue, to be discussed later in this viewpoint, is whether or not individual beliefs in willpower are problematic when an individual applies their own belief to others.

Even though the justice system does not operate on “proof” of the existence of free will, there has been a long history of open acknowledgment by senior members of the legal profession that *belief* in free will—the popular version described above—underpins the entire enterprise. Famed law professor Meir Dan-Cohen notes that criminal law is clustered around “*the Kantian conception of the responsible human subject as the noumenal self, characterized exclusively by a rational free will unencumbered by character, temperament, and circumstance*” [19]. Various United States Supreme Court rulings clearly emphasize that free will is a fulcrum [20,21]. For example, in *Steward Machine Co. v. Davis, Collector of Internal Revenue*, the court stated that “*the law has been guided by a robust common sense which assumes the freedom of the will as a working hypothesis in the solution of its problems*” (*Steward Machine Co. v. Davis, Collector of Internal Revenue*) [22].

Given that rational free will is uncoupled from individual differences and lived experiences (i.e., character and circumstance, as mentioned by Dan-Cohen, above), the courts also assume that the capacity to make such choices generally sits on a level playing field. That is, courts presuppose that populations funneled into the criminal justice system maintain a standardized ‘power’ or level of free will, a light switch effect that is knowingly and intentionally powered up to initiate law-abiding actions and, as needed, restrain criminal impulses. Critically important to the discussions below, the assumption of free will and willpower underpin, justify, and make normative, the infliction of punishment [11,23,24,25].

In 1846, Chief Justice John Gibson of the Pennsylvania Supreme Court opined that moral insanity might be the product of “*an unseen ligament pressing on the mind, drawing it to consequences which it sees, but cannot avoid*.” Yet, Gibson feared the implications of his proposition, stating further that even if legitimate, the wide application of a broken ligament excuse could cause chaos: “*If juries were to allow it as a general motive operating in cases of this character* [serious violence] *its recognition would destroy social order as well as personal safety*” [26]. Justice Gibson’s statement portends the fear-based marginalization of science, lest it excuse the behavior of too many.

By and large, the courts treat all individuals as normal, while acknowledging that there are rare exceptions to the free will assumption. These exceptions might include a defendant who presents with objectively measured brain *damage* (i.e., clear tissue destruction) or obvious and significant neurocognitive developmental impairments. Without evidence of gross structural damage, the courts, which is to say, prosecutors, judges, and juries, are very resistant to defense claims that individual differences in brain *function* (e.g., neurotransmitter turnover, frontal cortex use of glucose) influence intent, mens rea, and the capacity to choose [7,27,28].

## 3. System Maintenance Position

“*Current get tough proposals are hardly very tough or immoral. In fact, they are arguably quite just*”

[29] Stephen J. Morse, 1975.

Courts still operate under the free will assumption and exposure science, and neurobiology, outside of some minimal exceptions, remain at the fringes of criminal justice. Retribution remains the dominant mindset [30]. Current research suggests that presenting evidence of mental illness, no matter the severity, has very little influence on mitigating the harshness of sentencing [31]. Offerings of alternative pleas such as ‘guilty but mentally ill’ sound good on paper, but mostly translate into the same harsh punishment (i.e., no difference in sentence length), housing within deplorable penitentiary conditions, and minimal attention to actual mental health care [32].

Public health scholars and practitioners make important contributions in raising awareness of the many problems associated with mass incarceration, including the consequences to individuals, families, and communities that carry the burden [33]. However, there has been limited scrutiny of the ‘upstream’ ways in which legal scholars justify the overall system. How is the system maintained in the first place? Scholars who justify the system—concluding that criminal blame and punishment in the current system is both coherent and fair—lean on the word ‘normative’ and argue that responsibility in the legal frame is largely political and moral, and not a scientific matter [34]. That is, rationality is the fulcrum of criminal responsibility, and rationality in the legal context is ‘normative’ in that it is understood by the generalized values, principles, and morals held by society and its representatives.

The law assumes that almost all persons, with very rare exceptions, are rational actors that understand how to act with good reasons, and to refrain from acting if it will otherwise cause potential harm(s). Since the legal bar of rationality is set very low, it is presumed to be an unexacting task to abide by the law—perhaps more difficult for some than others, but relatively easy on the whole. The law makes room for its own non-scientific and non-medical construct of ‘insanity,’ wherein minimal rational capacity is virtually absent.

The McNaughten Rules of 1843, which still hold sway in many Global North countries, include four elements to arrive at a legally legitimate ‘insanity’ defense: i. defect of reason, ii. disease of the mind, iii. not knowing the nature and quality of the act, and iv. not knowing that what one is doing is wrong. In order to arrive at elements ii to iv, the defect of reason must be shown. Those who justify the system argue that only a minimal amount of reason and rational “power” is required. The take-home message, one that largely avoids criticism from public health, is that “*criminal law theory and neuroscience, or human biology more broadly, can stay in their respective silos. Anglo-American criminal law must assume free will even if the assumption is no more than folk psychology*…*because the vast majority of persons in Western societies believe in free will*” [35].

One prolific legal scholar, Stephen J. Morse, has been vigorously defending the system for some fifty years. Morse argues that unless there is clear evidence that the minimal levels of expected rationality have been lost, an entire collective of biopsychosocial causes does not equate to excuse. It is important for public health scholars to analyze the language used by those who justify the system. Here we have a snapshot of Morse’s system defense taken in the mid-1970s, at the front end of the “get tough” policies that fostered mass incarceration and major increases in life without parole sentences:

“*Broadening the class of persons who are considered not responsible for their behavior seems dangerous to public order and disrespectful to the personal dignity of individuals…limiting the defenses of non-responsibility would most benefit society. I propose that we constantly seek to limit these defenses in order to make clear to individuals that society views them as responsible persons who are in control of their lives and who are accountable for their actions. Self-control and moral behavior are always achieved with difficulty; but even so, the law’s presumption of responsibility will encourage the internalization of [moral] control…*

*Poverty is neither a sufficient nor a necessary cause of crime. Poverty may make the choice to obey the law more difficult, but the poor have choice whether to engage in crime, and the majority choose to obey the law*”.

[29]

On persons with mental disorders, Morse argues that court decisions are moral, not scientific: “*The law should not treat mentally disordered persons significantly differently from non-disordered ones because there is little persuasive scientific evidence that the former have significantly less control over their legally relevant behavior or are more predictable than the latter…*

*…even if such causes of crazy behavior are discovered, however, the discovery should not itself compel the conclusion that crazy people are diseased and not responsible for their behavior…it should be recognized that the ultimate decisions are moral and social and that special treatment rests on strong intuition and not on a scientific rationale*”.

[36]

System defenders argue that “*the person capable of rational conduct is at fault if she does not exercise her general capacity for rationality…prior events for which agents had no responsibility were part of the causal chain that led to the conduct in question. But so what? Whatever causal chain may have been operative, some agents are rational and some are not; some face hard choices, and some do not…determinism is no internal threat at all to the coherence and consistency of holding people responsible and blaming them*” [37]. Taken as a whole, evolving neuroscience and behavioral genetics really do not matter that much, they claim: “*Discovery of genetic or of any other physical or psychosocial cause of action raises no new issues concerning responsibility, and discovery of such causes does not per se create an excusing or mitigating condition for criminal conduct or any other type of behavior…there is good reason to believe that most addicts are responsible for seeking-and-using behavior and for other immoral or criminal activity related to addiction*” [38].

Defenders of the system maintain that reliance upon folk psychology is an absolute necessity: “*The act of punishment not only decrees social condemnation but incapacitates because it assumes that those who acted wrongly will choose to do so again unless they bear the full weight of shame society assigns to criminal conduct. By necessity, behavior judged criminal is thusly considered by its nature to be willful in a folk psychological sense*” [39].

Here, in this context of the claimed ‘necessity’ of folk psychology, a brief sortie into the topic of obesity may be worthwhile. Obviously, obesity is distinct from criminal behavior, and persons living with obesity do not represent a danger to society. However, the underlying and enduring social judgements of personal responsibility, agency, willpower, and blameworthiness, vis à vis the science of obesity, are highly relevant.

## 4. Obesity, Blame, and ‘Moral Fiber’

The prevailing (unscientific) view that obesity is a choice and a moral failing is based on the same willpower-based folk psychology [40] that is claimed to be a necessity by justice system defenders. Surveys in the United States indicate that a large number of adults believe that obesity is a willpower or self-control problem [41,42]. The false “commonsense” idea that obesity can be remedied simply by exercising willpower is “cemented in the minds of the general public and much of the medical profession” [43]. In a recent international scientific consensus paper, experts underscored that the folk psychology view of obesity needs to be dismantled: “Challenging and changing widespread, deep-rooted beliefs, longstanding preconceptions, and prevailing mindsets requires a new public narrative of obesity that is coherent with modern scientific knowledge” [44].

People who hold stronger beliefs about free will are more likely to ‘blame’ persons living with obesity or schizophrenia for the condition—likely because of the perception that the person is not tapping into their personal agency to otherwise ‘control’ their condition [45]. Similar findings have been reported in the case of addiction, wherein greater belief in a personal agency framework (as opposed to addiction as a neurobiological disease) is associated with a higher moral responsibility attribution [46]. In mock trials, overweight subjects are more likely to be considered blameworthy and punished, and female defendants who are overweight or obese are perceived to be a repeat offender, and more likely to be found guilty [47,48]. Thus, folk psychology beliefs justify punishment.

Scientific findings are challenging the neoliberal narratives of ‘personal responsibility’ and ‘agency’ that have been historically directed persons living with obesity [49,50]. Only in recent years have scientists turned from harmful narratives that otherwise situate people living with obesity and type II diabetes as ‘blameworthy’ for not having used their agency, willpower, and free will, to make healthy choices [51,52]. Public health practitioners understand that elected officials (often out of fear of “nanny state” accusations) and corporations favor interventions that require high levels of personal agency, and that these interventions not only have limited effectiveness, but they may indeed magnify existing socioeconomic inequalities [53,54].

The absurdity of this historical but ‘normative’ moral fiber stance can be visualized by examining the sudden and significant rise in global obesity over the last four decades. Upholding the blameworthiness and ‘moral fiber’ argument of obesity requires belief that there was a sudden loss in willpower among the global population. To take this position one must ignore the scientific reality that obesity has little to do with willpower and much to do with the intersection of neurobiology, gut microbes, adversity, and the endocrine system, all operating in a socioeconomically influenced obesogenic environment where hyperpalatable ultra-processed foods dominate [55].

In the past, much has been written about obesity and agency. Some argue that the person who lost weight in a particular program and kept it off tapped into their agentic skills. Perhaps this is so, but what does it say about the person who tried the same program and did not lose weight? Did they not think enough positive thoughts? Were they not optimistic enough? Did they not work as diligently at their goal-directed behavior? Were they harmed by the pressure of being in a program where willpower and agency was assumed, by practitioners, to be the difference between success and ‘failure’? Biologist Robert Sapolsky, who collated scientific evidence indicating that free will is an illusion [56], argues that the person who successfully loses weight can be thankful for the brain architecture that matched up with the intervention and helped to facilitate goal-directed behavior [57]. What we know for sure is that the person who was ‘unsuccessful’ should not be judged or considered blameworthy, but this is the precise response that is fostered by folk psychology.

Individuals are more likely to perceive, or *notice*, that their intent precedes action and results, which could be why agency is so often attributed to freedom of the will [58]. Free will skeptics accept that agentic skills and competencies exist. However, they contend that their development and use in addressing life challenges is limited (or constrained) at the individual level. This is distinct from a fatalistic or even pessimistic view. It is, however, a view that dismantles the blame directed at persons living with obesity and other folk psychology narratives that otherwise suppose that the intervention non-responder “does not want to be well.” Meta-analyses reveal that positive psychology interventions (even if we set aside poor evidentiary quality) have small to modest effect sizes [59,60]. Among other things, this suggests that interventions bump up against agency constraints and the realities of brain architecture.

## 5. Emerging Science

“*The ever more labored attempts to rescue free will from the steamroller of science take on the aspect of a carnival show*”

Joachim I. Krueger and David J. Grüning, 2024 [58]

Unprecedented advances in neuroscience, polygenic risk, neuromicrobiology, and the public health exposome—aided by related ‘omics’ technologies, and the massive growth in research under the developmental origins of health and disease (DOHaD) rubric—are assembling around the legal profession [61]. Assumptions of ‘agency’ look less impressive when paired with cutting-edge genome research showing the extent to which genetic loci are connected to major personality traits and the interactions between those traits [62]. Neanderthal DNA resonates over millennia, influencing whole brain functional connectivity patterns, dopamine signaling, and social cognition in the modern brain [63,64]. Indeed, Neanderthal-derived alleles may predict neurodivergence in modern humans [65]. The legal cradle of universal levels of ‘rationality’ is challenged by new research which demonstrates the significant degree to which cognitive rationality is determined by genetics [66].

The effect size of polygenic indices on childhood externalizing behavior has recently been reported to be on par with well-established risk factors such as childhood maltreatment and peer victimization [67]. Multi-omics studies (i.e., the combined use of genomics, epigenomics, transcriptomics, and/or metabolomics) are establishing links between biological markers, complex traits, and childhood neurobehavioral disorders and aggressive behavior [68,69]. Research continues to document the prenatal, perinatal, and even transgenerational influence of adversity in childhood and adult risks of neuropsychiatric disorders, addiction, and antisocial personality [70,71,72]. Volumes of preclinical studies illuminate bio-mechanistic pathways between adversity and cognitive behavioral outcomes, including altered mitochondrial function [73]. Mitochondria, which appear to be involved in impulse control, might be considered the psychobiological “power” component of what is assumed to be “will” [74,75,76]. The gut microbiome is also an “unseen” factor in behavior deemed criminal [77,78].

Of course, it is true, as the system defenders point out, that the vast majority of persons living with poverty do not engage in criminal behavior. However, research continues to point toward poverty as a provocateur of crime [79]. In a recent Brazilian study that examined 22 modifiable individual and family exposures assessed in childhood (5–14 years old) and criminal conviction at a 7-year follow-up (13–21 years, *n* = 1905, and 76% retention rate), poverty at baseline was the only modifiable risk factor significantly associated with subsequent conviction. The authors conclude that preventing children’s exposure to poverty would reduce nearly a quarter of subsequent criminal convictions [80].

## 6. Public Health Quarantine Model

“*If society has a right attitude toward the subject, if it has sympathy, imagination and understanding, it will isolate these victims, not in anger but in pity, solely for the protection of the whole*”

Clarence Darrow, 1924 [81]

Darrow argued that retributive punishment was underpinned by provocation of hate, stirred on by media, prosecutors, and ancient ideas of ‘evil’: “*Free will is a myth. What to do about those who break laws is something that scientists, not jurors, should determine. Most of them* [criminals] *never had a chance. We never try to punish those we like. First, we must hate them*” [82]. Given Darrow’s position that criminal behavior should not be met with retribution and blame, he was often asked how society should go about protecting itself. With moral responsibility off the table, what would reimagined public safety look like? Darrow’s vision was akin to the public health model of infectious disease, wherein the rights of the individual can, if conditions warrant, be subordinated to the public as a whole.

Separation from society was, according to Darrow, already punishment enough. It would be up to a diverse group of people, using wisdom and presumably science and evidence-informed practices (absent hate and fear), to determine readiness for reentry: “*If I had my way, a jury would decide nothing except guilt or innocence of the prisoner and there would be no sentences. The* [person] *who has been convicted would be isolated in a hospital, under the close supervision of a board of wise, tolerant, and humane, men and women*” [6]. He used the infectious disease model to illuminate the disinterest in wellness and arbitrary nature of carceral punishment: “*If a lawyer discovered a man with typhoid fever he’d give him 60 days in jail, let him out at the end of 60 days, even if he were worse, because his time was up. Or, if he were well at the end of 10 days, keep him there until his time was up just the same*” [83].

Darrow did not develop a full treatise on his quarantine idea, nor did he expand upon the ethical conundrums it might present. His main thrust was that a quarantine model was distinct from harsh, retributive carceral punishment by removal of the underlying mindset of free will assumption, hate, and fear: “*As a rule, lawyers are not scientists. They have learned the doctrine of hate and fear, and they think that there is only one way to make men good, and that is to put them in such terror that they do not dare to be bad*” [84]. Only in recent years has the quarantine model been evaluated through the lens of public health ethics. This is a vital step; as public health professionals understand, any discussions of society’s right to protect itself, individual liberties, and the ultimate aims of the justice system, require thoughtful consideration of ethics.

**Figure 1:**
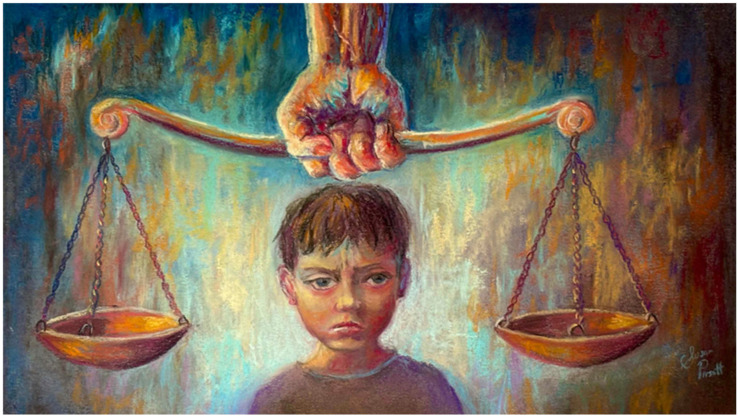
“So what?” Defenders of system argue that even though advances in biopsychosocial sciences might show that it is harder for some people to follow the law, in the courtroom it should have little influence in excusing conduct (Artwork by author, S.L.P.).

The public health quarantine model of criminal justice, thoughtfully developed by scholars Derk Pereboom and Gregg D. Caruso, imagines a criminal justice system wherein the primary function is prevention, one that actively addresses the social, environmental, and developmental origins of crime (Figure 1). It is underpinned by Darrow’s skepticism of willpower-based moral agency. According to its contemporary advocates, the public health quarantine model assumes that “what we do, and the way we are, is, ultimately, the result of factors beyond our control, and, because of this, we are never morally responsible for our actions in the basic desert sense” [85].

**Figure 2:**
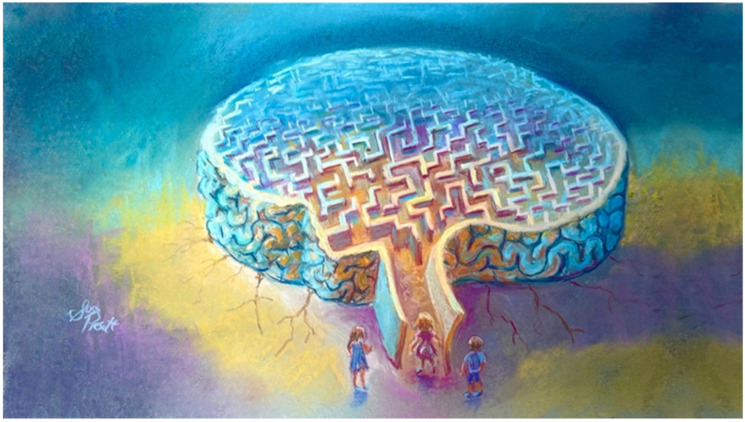
The non-retributive public health quarantine model emphasizes preventive approach that actively addresses the social, environmental, and developmental origins of crime (artwork by author, S.L.P.).

While acknowledging biology, the model does not translate into the idea that crime is a biological “disease.” Rather, it emphasizes overlaps between the social determinants of health and the social determinants of behavior deemed criminal. It looks for the many ‘Broad Street pumps’ (i.e., the many sources of justice involvement) and attempts to remedy the causes of the causes. For example, substance use may have caused imprisonment, but trauma and neurobiological factors may cause an increased risk of use [86,87]. Many of those upstream causes may be rooted in various sources of adverse childhood events, and the adverse events experienced by parents (Figure 2). Adverse events are often an intergenerational problem. Consider that children whose parents reported four or more of their own adverse childhood events are almost twice as likely to be arrested at age 25 or younger compared to parents who reported a general absence of (zero to one) adverse childhood events [88].

The public health model emphasizes that a large number of justice-involved juveniles and adults experience prior victimization. In other words, there is acknowledgement of a ‘contagion’ in which victimization begets new victims [89]. The history of victimization among the accused has been observed across different groups, geographies, cultures, ages, and types of crimes [90]. However, the public health model is also concerned with cumulative positive childhood experiences (safe, stable, and nurturing relationships) which are beyond the absence of adverse events. Positive childhood experiences are associated with lower risks of delinquency and risk-taking behavior, higher levels of self-control, and better mental health in adulthood [91].

The model acknowledges that public health quarantine, whether it is based on highly contagious infectious disease or criminal behavior, can be ethically backed by government force. However, the level of restraint must be legitimate. In a case of infectious disease, it is not ethically legitimate to treat the person in harmful ways that are beyond the need for social sequestration and threat neutralization. The same principle of least infringement applies to justice-involved persons, and retributive “just deserts” are off the table. The level of restraint and endurance of incapacitation is governed by the potential of dangerousness and harm, not by ideas of consequentialist deterrence. In this model, any incapacitation demands concern for rehabilitation and wellbeing; if, over time, it is determined that an individual is beyond rehabilitation, and public safety requires their indefinite confinement, the quarantine model provides no justification for misery, and the only level of restraint is that required to guard against legitimate danger [92].

In the context of public health, legal policy, and mandatory infectious disease quarantine (the latter distinct from general advice to “isolate”), most US states maintain enforceable statutes that would apply to an individual [93]. Despite borrowing from public health ideas of infectious disease quarantine, and utilizing the term public health in a front-facing direction, discourse concerning the public health quarantine model of criminal justice remains almost exclusively within the silo of philosophy. Even a basic Google Scholar search of the term “public health quarantine model” reveals an absence of critical analysis by experts within public health and/or collaborators from the fields of prevention science and whole-person health. We suspect that the majority of these experts would be sympathetic to the ideas of reducing unjustified punishment and shifting the focus toward prevention and early-life investments. System defenders might crow about modest declines in US prison populations over the last decade, but public health and allied professions would surely not be content with a current decarcerating pace that would take until 2098 to arrive at incarceration rates that existed in the early 1970s [94].

Where the quarantine model becomes tricky is the requirement of preventive detention based on evidence of dangerousness or potential harms to society. In Caruso’s version of the criminal quarantine, “*only those who pose a serious threat to society can be incapacitated on the grounds of self-defense and the defense of others*”, and it is recommended that “*the burden of proof* [is] *on the state to establish, at regular intervals, that the threat posed by an offender warrants continued incapacitation*” [95]. As critics point out, for those who are sequestered, the model is dependent upon a high level of accuracy in determining future harms [96]. Determinations of contagion risk in most infectious diseases is far less complicated than assessing future behavioral dangerousness. Who is going to be in control of determining risk-based detention? Will it be wise women and men, as advocated for by Darrow? What kind of evidence and criterion will be used to enact and lift detention? Caruso resists the idea of preemptive incapacitation [97], but would the model, if implemented, lead to the confinement of persons who have not committed crimes?

Undoubtedly, the model will require risk assessment tools, and hopefully better instruments than those currently employed by the system. Traditional and algorithm risk assessments, especially for violence, are prone to racial biases [98,99]. Machine learning offers promise, yet the sources of upstream information, such as prior arrest records, might be not be a proxy of proven crime, and instead be a proxy of racial discrimination [100]. If structured professional judgement tools such as Historical Clinical Risk Management-20 V-2, the Static-99, or the Violence Risk Appraisal Guide, were evaluated using the generally accepted values for the diagnostic accuracy of tests in healthcare settings, the predictive accuracy (around 64–70%) would be considered poor to fair [101]. Despite this weakness, such judgement tools are already in widespread use in parole and bail assessments. Recently, it has been argued that should the research become more robust, supplementation of judgement tools with neuroimaging and electroencephalogram findings [102], along with biological markers [103], and microbiome signatures [61], will elevate predictive accuracy.

In the public health quarantine model, questions of autonomy and dignity in the context of human rights emerge. Contemporary scholars who side with Darrow, such as lawyer Richard Oerton, also argue that the abandonment of prescientific willpower beliefs must be accompanied by the assurance of autonomy [10]. What about dignity? Does the suggestion that there are major constraints on agency damage dignity and facilitate dehumanization, as some claim [104], or is the opposite true—is human dignity undermined by continued propagation of free will, willpower, and near-unlimited agency assumptions [9,10]? In the justice context, here is Morse’s view of free will, dignity, and deservedness of suffering:

“*Except in extreme cases justifying a total defense such as insanity, duress, necessity, or self-defense, it is simply not that hard to obey the law…the great value of this position, placing the burden of persuasion on those who believe it is hard to obey the law, is that it treats people with greater respect and dignity than the opposing view, which treats them as helpless puppets buffeted by forces that rob them of responsibility for their deeds…there is simply no defendant, no matter how privileged, for whom a convincingly sad tale cannot be told… the strong norm of equality should not yield to the common and dignity-robbing assumption that differences in responsibility among the responsible are so great…causing people to suffer for the crimes they have committed does not offend me. They suffer because they deserve it*”.

[105]

The latter portion of the quote should ring alarm bells among public health practitioners. Critics argue that Morse brings a strawman into the dignity and differentness claim. First, even the staunchest advocates of determinism, such as Sapolsky, do not claim that humans are helpless puppets (on the contrary, he advocates for a society that focuses on early-life development of empathy [106]), and second, how could one party be robbed of dignity when no one, not the ‘upstanding’ citizen or the convicted, is considered morally blameworthy [107]? Sapolsky argues that although “there is a certain appealing purity” to Morse’s claim of dignity, acknowledging the neurobiological reality of significant individual differences is “vastly more humane than moralizing them into being sinners” [108]. Perhaps discussions of dignity are much easier for the person in elite academia than they would be for the person who, with evidence of an underactive prefrontal cortex [109], high tissue levels of lead from Flint-like water [110], low levels of omega-3 [111] and various nutrients associated with food insecurity [112], polygenic loci linked to neurodivergent behavior [113], dysbiotic microbiome [114], blunted resting heart rate [115], mitochondrial dysfunction [116], and having endured the ‘hits’ of multiple adverse childhood events [117] and victimization by a ‘neurotypical’ society [118,119], suffers in wholly undignified carceral conditions.

In the public health quarantine model, Caruso challenges the idea that retribution and just deserts preserve dignity at the all-of-society level. He argues that the public health model provides dignity for all, and affords no justification for dehumanization and cruelty in association with quarantine. Caruso describes the retributive conception of human dignity among system justifiers as the authority figure who practices corporal punishment, and while doing so claims that it is only out of respect for the recipient as a morally responsible agent: “*This is a perverse notion of human dignity and should never have gained traction in the first place*” [95].

Here, public health practitioners might be interested in content analysis of law journals wherein harsh carceral conditions, as part of the linkage between legal and public expectations of moral responsibility, and associated ‘needs’ for shame and suffering, are described without demur [10,11]. Beyond objection-free discussions of carceral suffering, there is a large body of academic discourse, mostly found in law journals, wherein advocates for overt public shaming of criminals (distinct from fines or community service, which largely bypass shame) that seems disinterested in the dignity of justice involved persons. Only a few scholars argue that both overt public shaming and the typical conditions of incarceration are at odds with a society that treasures dignity for all, and such practices are ultimately underpinned by the development of specific attitudes and emotions directed at the out-group member—hatred, anger, fear, and indifference to cruelty [120].

Since people are less likely to punish an in-group member [121], an out-group member must be vilified. Few law scholars discuss the extent to which the entire criminal law enterprise works to create an out-group that is perceived to be deserving of punishment [122]. It cannot be assumed that the “fraudster” white/grey-collar criminal who grew up with *economic* privilege escaped adverse child events or does not have brain architecture that increases vulnerability [123]. On the contrary, twin research shows that the unique causal pathways to white-collar criminal behavior are largely genetic, with a small contribution of shared environmental influences [124]. Separate studies show significant differences in brain structure/function in white-collar crime [125,126], and a variety of genes, hormones, and neurotransmitters have been linked to financial risk-taking [127]. Of course, greed as a pathway to justice involvement has social and developmental inputs [128], which will require a strong preventative focus under the model.

At the same time, the public health quarantine model cannot escape the fact that retributive justifications for punishment are deeply embedded in westrern culture and legal systems. Moreover, the scientific study of punishment tells us that retributive impulses are also part of our biological and psychological make-up [129]. For example, survey respondents might claim that they strongly support criminal justice reform, but that claim is softened when respondents are confronted with vignettes or real-life cases of jarring crimes. Experiments show that there is an emotional boost, or what some term “the joy of punishment”, when subjects engage in or witness the punishment of transgressors [56,130]. Research subjects are particulalry motivated to seek punishment if it is associated with an understaning (by the transgressor) of the reason for the suffering, even if it does nothing to change future behavior [131]. It is likely that the “joy of punishment” extends to the long-term social labeling of justice-involved persons. Research documents the detrimental effects of labeling; indeed, professionals working in the criminal justice system have contributed to labeling [132]. Without adequate care, confinement under a public health quarantine model runs the risk of contributing to labeling.

The public health quarantine model cannot ignore the social demands of reciprocity. Across time and cultures, the concept of justice—described as “the reciprocal quality of relationships that obtain between people for their mutual wellbeing”—may be a basic human need [133]. While victims largely prefer shorter sentences and greater investments in treatment/rehabilitation [134], they (and large segments of society attuned to the consequences of victimization) may recoil at a public health quarantine model that seems to sidestep punishment. The model invites serious questions, including the idea that society will abandon support of a government that appears unwilling to mete out some degree of expected “justice” (i.e., punishment), even for non-violent offenders [135]. When victims experience harm but perceive that justice has not been served—or that the principles of fairness and reciprocity have been violated—they may suffer ongoing psychological distress and a deep erosion of trust in others and in society [136]. Does the public health quarantine model fully capture potential harms associated with social perceptions of unfairness? Related to this is the larger question of the degree to which society views free will “incentives” (i.e., avoiding punishment) as part of whole-of-society flourishing.

In sum, implementation of the public health quarantine model will likely run into problems because it is a significant departure from the public’s view on deserts and the expectation that the government will punish offenders. Such major departures from public expectations present a problem of government legitimacy [137,138]. The bridge between the public health quarantine model as an aspirational concept and reciprocity-based justice might be found in non-retributive concepts such as restorative justice. Described in detail elsewhere [139], restorative justice emphasizes healing by fostering respectful, inclusive, and humane dialogue. It provides victims and affected communities with a meaningful voice, creating space for accountability and recognition of harm. The goal is to bring together those who caused harm and those who have been harmed—whether individuals or communities—to work toward understanding and, where possible, reconciliation. While more research is needed, the approach has been linked to benefits for all parties, including lower recidivism [140,141].

## 7. The Fallacy of Phineas

Finally, although the public health quarantine model is not focused on a piecemeal expansion of categories of excuse for criminal conduct, its determinations of isolation will require evidence-informed approaches. Our concern in this regard is the historical demarcations between “organic” disease and so-called “functional” disorders. History demonstrates that assumptions run amok in the area of “functional” disorders. Free will exceptions are made when an individual has significant “organic disease” and/or tissue destruction in the brain, and anything less is often folded into the increasingly meaningless term “psychosomatic.”

The famous case of railroad foreman Phineas Gage, who survived a tamping rod through his skull, is oft used to convey the idea that damage to a frontal lobe can rapidly upend longstanding features of personality and self-control. In the months after the 1848 injury, Gage’s physician noted that the once thoughtful and highly regarded worker, a person who had a history of planning and concern for future consequences, lost the balance between his “intellectual faculties and animal propensities” [142]. Post-injury, Gage “was no longer Gage.” Contemporary workplace injury (i.e., frontal lobe) cases support the observations of Gage’s physician—i.e., long-term executive functioning deficits, poor social awareness and self-monitoring skills, defective abstraction, cognitive inflexibility, and deficits in planning [143].

Frontal lobe damage (e.g., through tumor and other mechanisms) has been noted as a potential driver of relatively sudden onset criminal behavior and ‘acquired sociopathy’ [144,145]. If a person with an obvious traumatic brain injury became justice-involved, juries would likely lean toward leniency and a perception that the defendant’s free will had been compromised [27]. This might be especially true for jury members with a higher education background in biology and/or behavioral sciences [146,147]. Put simply, evidence of so-called structural damage is easier to visualize, both through neuroimaging and in the imaginings of the triers of facts.

Some worry that the abandonment of moral responsibility would allow someone with “*no apparent brain damage—think Bernie Madoff—who showed no propensity for future violence*” to avoid public health quarantine [148]. First, the quarantine model is based on potential harm, not violence alone, and there is little doubt that white-collar crimes, especially at the scale of Madoff, cause massive trauma and social harms [149]. Second, given that recidivism rates of white-collar crime are significantly higher than violent crime (twice as many of the former are convicted of a new crime and reimprisoned within three years vs. the latter [150]), and given links between firm personality traits and white-collar crime [151], the ‘Madoffs’ might be most at risk for long term confinement. Third, and perhaps most importantly, the assumption that significant legally relevant alterations in behavior are dependent upon “apparent brain damage” and tissue destruction, rather than alterations in biophysiology, is a fundamental biolegal error that is termed here as the fallacy of Phineas.

Individuals with significant brain pathology (e.g., documented traumatic brain injury) still find themselves swept into the ‘normative’ funnel of criminal law, judged to be well capable of making free choices, and placed in typical carceral settings, if not death row [152,153]. Yet, our concern here is with the *necessity* of visualized destruction or growth, such as a tumor [27]. Among other subdisciplines, neuromicrobiology is erasing false lines between structure and function. There is no need for tissue destruction in order for critical “structures”—such as the intestinal barrier and blood–brain barrier—to be more porous and allow for a cascade of physiological events, including immune cell involvement, that otherwise disturb cognition and behavior [154,155]. These structural alterations may not be evident via traditional neuroimaging or gastrointestinal testing, but exist nonetheless. Various environmental exposures would be enough to make a contemporary Gage, cognitively and behaviorally, “no longer Gage” [77,156].

The fallacy of Phineas, with its insistence upon tissue destruction, obscures the explanatory power of biophysiology and allows for the continued criminalization of mental illness. It also trivializes the critical neurobiological differences between neurodivergent and neurotypical populations. Those differences, which are not based on “apparent brain damage”, equate to heightened risk of adverse childhood events (likely because the neurotypical blame the neurodivergent person for not tapping into their inhibitory “willpower”) and subsequent justice involvement [157].

## 8. Conclusions

“[What] *if, for example, society itself were responsible for any deprivations or degradations that the actor had suffered, society might not be entitled to condemn that actor*”

Judge David L. Bazelon, 1976 [158]

Writing in the Southern California Law Review some fifty years ago, US Circuit Court judge David Bazelon suggested that accumulating evidence on poverty and structural inequities were demonstrating that society writ large is playing a role in criminal behavior, and as such, undermines its right to punish an afflicted person. Similar to Darrow, his ‘big minority view’ did not permeate the legal profession. Today, advances in biopsychosocial and environmental sciences continue to demonstrate the ways in which the interacting forces of heredity, lived experiences (both positive and negative, and especially early-life), various exposures, and brain architecture, influence cognition and behavior in the here and now.

If the foundations and girders justifying moral blame and punishment are illegitimate, as famed attorney Clarence Darrow contended a century ago, then society needs to consider a more public health-oriented lens toward criminal justice and incapacitation. Darrow’s idea was a shift in focus toward the social determinants of health and a non-punitive confinement that would resemble public health’s approach to highly contagious infectious diseases. This idea has been expanded upon by scholars and shaped into a contemporary public health quarantine model.

To date, discussions of the model have mostly been confined to philosophy journals and outlets external to primary public health discourse. We provided a basic introduction to the quarantine model. We placed it central to other vigorous discussions; these include increasingly louder argumentation related to constraints on agency, and the ways in which legal scholars justify the status quo of retribution, punishment, and the marginalization of science. While public health has a strong record of highlighting the harms of mass incarceration, research and commentary has generally not been directed at the prescientific “normative#x201D; underpinnings of the system.
